# Potential regulatory genes of light induced anthocyanin accumulation in sweet cherry identified by combining transcriptome and metabolome analysis

**DOI:** 10.3389/fpls.2023.1238624

**Published:** 2023-08-16

**Authors:** Yao Zhang, Chaoqun Chen, Yiling Cui, Qinsong Du, Wenjing Tang, Wenlong Yang, Guanqiong Kou, Wanjia Tang, Hongxu Chen, Ronggao Gong

**Affiliations:** College of Horticulture, Sichuan Agricultural University, Chengdu, China

**Keywords:** sweet cherry, anthocyanin, Shading, WGCNA, 4CL

## Abstract

Anthocyanins exist widely in various plant tissues and organs, and they play an important role in plant reproduction, disease resistance, stress resistance, and protection of human vision. Most fruit anthocyanins can be induced to accumulate by light. Here, we shaded the “Hong Deng” sweet cherry and performed an integrated analysis of its transcriptome and metabolome to explore the role of light in anthocyanin accumulation. The total anthocyanin content of the fruit and two of its anthocyanin components were significantly reduced after the shading. Transcriptome and metabolomics analysis revealed that *PAL, 4CL, HCT, ANS* and other structural genes of the anthocyanin pathway and cyanidin 3-O-glucoside, cyanidin 3-O-rutinoside, and other metabolites were significantly affected by shading. Weighted total gene network analysis and correlation analysis showed that the upstream and middle structural genes *4CL2, 4CL3*, and *HCT2* of anthocyanin biosynthesis may be the key genes affecting the anthocyanin content variations in fruits after light shading. Their expression levels may be regulated by transcription factors such as *LBD, ERF4, NAC2, NAC3, FKF1, LHY, RVE1*, and *RVE2*. This study revealed for the first time the possible role of *LBD, FKF1*, and other transcription factors in the light-induced anthocyanin accumulation of sweet cherry, thereby laying a preliminary foundation for further research on the role of light in anthocyanin accumulation of deep red fruit varieties and the genetic breeding of sweet cherry.

## 1 Introduction

Various kinds of plants are growing on the earth, and they show rich colors because they are rich in different pigments. These colors not only meet the needs of the plant’s own growth and reproduction, but also bring visual enjoyment to people. Among all the colors, red, blue, and purple are mainly affected by anthocyanin concentration (Rao et al., 2021). Anthocyanins are important secondary metabolites in higher plants. They belong to the flavonoid group and are water-soluble pigments (Zhou et al., 2022). Anthocyanins are usually synthesized in the cytoplasm and transported to vacuole storage through a complex transport system (Passeri et al., 2016). As one of the three major pigments of plants (Zhao et al., 2022b), anthocyanins promote pollination by insects to help plant propagation and help plants to resist low temperature, drought, salt, and other stresses to a certain extent (Alcalde-Eon et al., 2013; Zhang et al., 2019; Kim et al., 2022). With the deepening of research, increasing evidence from recent years indicates that anthocyanins also play an important role in maintaining physical health, such as anti-aging and vision protection properties and the reduction of the risk of diabetes and cancer (Gonçalves et al., 2021).

As the main environmental factors affecting plant growth and development, light, temperature, and water also affect the synthesis of plant anthocyanins (Li et al., 2019). In many studies, plants under different stress conditions will show varying anthocyanin contents compared with the counterparts under a normal environment. The anthocyanin content of red pears increased after white light exposure compared with double-layer paper bag coverage (Bai et al., 2019). After low temperature treatment at 16 °C, the upper leaves of the “Gala” apple showed obvious red spots (Song et al., 2019a). In the study of Jian-Ping An et al, apple seedlings after drought stress also showed an increase in anthocyanin accumulation (An et al., 2020b). Among these environmental factors, light is particularly important for anthocyanin synthesis in horticultural plants.

At present, the anthocyanin synthesis pathway has been examined in-depth in Arabidopsis, apple, strawberry, grape and other plants (Leong et al., 2018; Jiu et al., 2021; Liu et al., 2021; Nguyen et al., 2023). Anthocyanin is mainly synthesized by phenylalanine and malonyl-coenzyme A through a variety of enzymatic reactions involving structural genes such as *PAL*, *4CL*, *CHS*, *CHI*, *F3H*, *F3’H*, *DFR*, *ANS*, and *UFGT* (Jaakola, 2013). In addition to the influence of structural genes, the synthesis of anthocyanins is also controlled by many regulatory genes. The MBW ternary complex is the most widely studied regulatory factor in the anthocyanin synthesis pathway (Ma and Constabel, 2019). The said complex consists of the *R2R3 MYB*, basic helix-loop-helix *bHLH*, and *WD40* regulatory factors in plants and directly regulates various structural genes in anthocyanin biosynthesis. Anthocyanin synthesis is also regulated by other transcription factors such as alkaline leucine zipper *bZIP*, *WRKY*, and *NAC*, which function mainly through direct or indirect action on the MBW complex(An et al., 2018; Sun et al., 2019; Yue et al., 2022).

Similarly, the induction of light in plant anthocyanin biosynthesis is regulated by transcription factors. In red pear, *BBX16* can positively regulate the accumulation of photoinduced anthocyanins by activating *MYB10*, which significantly increases anthocyanin content in fruits after light restoration (Bai et al., 2019). *PybZIPa*, another important transcription factor, activates *PyUFGT* to participate in the light-induced anthocyanin accumulation in red pears by binding to the tandem G-box in the promoter (Liu et al., 2019a). Anthocyanin accumulation in apples is also induced by light (Yang et al., 2019). Studies have shown that *MdMYB1*, *MdBBX21*, *MdTCP46* and other regulatory factors are involved in anthocyanin accumulation (An et al., 2020a; Yang et al., 2021; Zhang et al., 2021), and the degree of anthocyanin accumulation in apples varies under different light intensities (Chen et al., 2019). In peaches, *PpHYH* activates the transcription of three *PpMYB10* gene clusters in the presence of cofactor *PpBBX4*, leading to anthocyanin accumulation in the peel under sunlight (Zhao et al., 2022a). Purple broccoli’s anthocyanin biosynthesis genes such as *PAL*, *4CL*, and *CHI* were significantly downregulated after shading, and the anthocyanin content decreased (Liu et al., 2020). These results indicate that light plays an important role in regulating anthocyanin biosynthesis in plants.

Sweet cherry (*Prunus avium L*.) is a perennial light-loving horticulture crop of the genus *Prunus* in the Rosaceae family, originating in the region between northeastern Anatolia, the Caucasus, and the Caspian Sea (Davis, 1975). Today, the fruit tree is grown globally in more than 40 countries with mild climates (Ceccarelli et al., 2020). Given their attractive appearance, delicious taste and rich nutritional value, sweet cherries are widely favored by consumers and have considerable economic value (Wei et al., 2015). At present, however, we know very little about the role of light in sweet cherries, especially red sweet cherry varieties. Thus, this study preliminarily explored the effect of light on anthocyanin accumulation in red sweet cherry varieties and its mechanism, thereby laying a foundation for further research on the role of light in anthocyanin accumulation in deep red fruit varieties and the genetic breeding of sweet cherry.

## 2 Materials and methods

### 2.1 Plant materials and treatment

“Hong deng” sweet cherry grafted on Prunus tomentosa was used as the test material in this study and was obtained from the sweet cherry test site in Buwa Village, Wenchuan County, Aba Tibetan and Qiang Autonomous Prefecture, Sichuan Province. Six sweet cherry trees aged 12 years with the same growth conditions and growth period were randomly divided into two groups of three trees each (i.e., three replicates). The test materials were uniformly managed from the end of March 2022 (initial flowering stage). In the experiment, the ‘Hong Deng’ sweet cherry fruit after shading bagging (SHD) was used as the treatment, and the normal light fruit without bagging (LHD) was used as the control. The sweet cherry fruit in the treatment group was bagged on April 25, 2022 (23 days after anthesis [DAA], fruit expansion stage), 300 fruits per tree were used for experimental treatment. Then, we randomly removed some of the fruit bags (RHD) the treatment group at the fruit maturity stage (55 DAA). Sampling began 23 DAA on the bagging day.15 sweet cherry fruits of the same size and which were free of diseases, pests, and mechanical damage were picked from the east, south, west, and north directions of the crown of each tree in each community according to a random sampling method, and were collected every 3 days until the fruits were ripe (63 DAA). A total of 45 fruits were collected per treatment in each period and a total of 11 period were collected. The samples collected each time were immediately placed in an ice box and brought back to the laboratory. After being photographed, the samples were quickly cut into uniform blocks, subjected to liquid nitrogen quick freezing, wrapped in foil, and stored in a refrigerator at -80 °C for use. Three biological replicates were set for each sample.

### 2.2 Determination of fruit color, anthocyanin content and composition

A total of 15 fruit samples with similar maturity and color at each stage were selected for each treatment. The skin color difference at three points on the equatorial line of fruits was measured by an automatic chromometer. The L*, a*, and b* values were also recorded.

Total anthocyanin content was determined by the hydrochloric methanol method described as follows (Zhang et al., 2018a). Weighed 0.2 g sample and add 5 mL HCl/methanol (1/99, v/v), shaked the mixture well, and stored at 4 °C away from light for more than 20 h, followed by ultrasonic extraction at 4 °C for 30 min. Then, the mixture was centrifuged at 8000 rpm and 4 °C for 10 min. Absorbance was measured at 530, 620, and 650 nm. Calculate A=(A530-A620)-0.1(A650-A620), and the result was expressed as the amount of nmol anthocyanins contained in each g of sample.

Anthocyanin components were determined by high performance liquid chromatography (HLPC) using the Agilent 1260 II liquid chromatograph on Comatex C18 column (250 mm × 4.6 mm, 5 µm) with a diode array detector (Chen et al., 2022). The detection wavelength was 520 nm, the column temperature was 30 °C, the sample size was 10 µm, and the flow rate was 1 mL/min. The mobile phase A was acetonitrile and the mobile phase B was 1.6% formic acid aqueous solution. The gradient elution procedure is as follows: 0–15 min 95%–85% B, 15–21 min 85%–72% B, 21–22 min 72%–60% B, 22–24 min 60%–40% B, 24–27 min 40%-95% B, and 27–30 min 95% B.

### 2.3 Metabolite assay

#### 2.3.1 Metabolite extraction

A tissue sample of 100 mg liquid nitrogen grinding was taken and placed in an EP tube with 500 μL of 80% methanol aqueous solution. The sample was subjected to vortex shock, ice bath for 5 min, centrifugation at 15000 g and 4 °C for 20 min. A certain amount of the supernatant was taken, diluted with mass spectrometry water until the methanol content was 53%, and centrifuged at 15000 g and 4 °C for 20 min. The supernatant was then collected and injected into LC-MS for analysis.

#### 2.3.2 Metabolite detection and analysis of chromatographic conditions

We used a HypesilGoldcolumn (C18, 100 × 2.1 mm, 1.9 μm) column to inject 2 µL of samples at a flow rate of 0.2 mL/min and column temperature of 40°C with an automatic injector set at 8°C. The positive and negative modes were adopted, with 0.1% formic acid as the positive mobile phase A and methanol as the mobile phase B. The negative mode mobile phase A is 5 mM ammonium acetate, pH 9.0, and the mobile phase B is methanol. The gradient elution procedure is as follows: 0–1.5 min, 98%–15% A, 2%–85% B; 1.5–3 min, 15%–0% A, 85%–100% B; 3–10 minutes, 0%–98% A, 100%–2% B; 10–10.1 minutes, 98% A, 2% B; and 11–12 minutes, 98% A, 2% B.

The mass spectrum conditions are as follows. The scanning range is 100–1500 m/z. The ESI source settings include the spray voltage of 3.5 kV; sheath gas flow rate of 35 psi; Aux gas flow rate of 10 L/min; ion transfer tube temperature (capillary temp) of 320°C; ion import RF level (S-lens RF level) of 60; Aux gas heater temp of 350°C; and polarities: positive, negative. The MS/MS secondary scans are data-dependent scans.

#### 2.3.3 Metabolome data preprocessing and metabolite identification

The raw file was imported into the CD 3.1 library search software for processing, and the retention time, mass–charge ratio, and other parameters of each metabolite were screened. The retention time deviation of 0.2 min and mass deviation of 5 ppm were set for the peak alignment of different samples, followed by peak extraction and quantification of the peak area. Then, the target ions were integrated, and the molecular formula was predicted by molecular ion peak and fragment ion and compared with the mzCloud (https://www.mzcloud.org/), mzVault, and Masslist databases. After standardized processing, the relative peak area was obtained. Compounds with CV greater than 30% of the relative peak area were deleted from the QC samples. Finally, the identification and relative quantitative results of metabolites were obtained. The data processing involved a Linux operating system (CentOS version 6.6) and the R and Python software.

#### 2.3.4 Statistical analysis of metabolome data

The KEGG database (https://www.genome.jp/kegg/pathway.html) was employed to identify the metabolites. In the multivariate statistical analysis, the metabolomics data processing software metaX was used to transform the data. Then, principal component analysis (PCA) and partial least square discriminant analysis (PLS-DA) were performed to obtain the VIP value of each metabolite. In the univariate analysis, the statistical significance (P value) of each metabolite between the two groups was calculated based on a t test, and the fold change (FC value) of the metabolite between the two groups was calculated. The default criteria for differential metabolite screening were VIP >1, P value <0.05, and FC ≥2 or ≤0.5. The cluster heatmap was drawn with the R-packet Pheatmap, and the metabolite data were normalized with a z-score. The KEGG database was also utilized to examine the function and metabolic pathway of the metabolites. When x/n>y/n, the metabolic pathway was considered enriched; when the P value of the metabolic pathway was <0.05, the metabolic pathway was considered significantly enriched.

### 2.4 Transcriptome sequencing

#### 2.4.1 RNA extraction and transcriptome sequencing library preparation

Total RNA was extracted using a total RNA kit (Beijing Tiangen Biotechnology Co., LTD., China), and the integrity and total RNA was accurately detected by the Agilent 2100 bioanalyzer. The starting RNA of the library was total RNA. mRNA with polyA tail was enriched by Oligo(dT) magnetic beads. The mRNA fragments were randomly interrupted by divalent cations in the fragmentation buffer. The first cDNA strand was synthesized in the M-MuLV reverse transcriptase system, then degraded by RNaseH. The second cDNA strand was synthesized in the DNA polymerase I system using dNTPs as raw material. The purified double-stranded cDNA was end-repaired, A-tail was added, and sequencing joints were connected. The cDNA of approximately 370~420 bp were screened with AMPureXP beads for PCR amplification, and AMPureXP beads were used again to purify the PCR products to obtain the A library.

After the library construction, the library quality inspection was performed. Once the library inspection was qualified, different libraries were employed for Illumina sequencing by pooling according to the requirements of effective concentration and target data volume. Consequently, a 150 bp paired end reading was generated. The basic principle of sequencing entails sequencing by synthesis described as follows. Add four kinds of fluorescently labeled dNTP, DNA polymerase and connector primers to the sequencing flow cell for amplification. When each sequencing cluster extends the complementary chain, each addition of a fluorescently labeled dNTP can release a corresponding fluorescence. The sequencer will capture the fluorescence signal and convert the light signal into a sequencing peak through a computer software, thereby obtaining the sequence information of the fragment to be tested.

#### 2.4.2 Transcriptome data analysis

Image data measured by high-throughput sequencers were converted into sequence data (reads) by CASAVA base recognition in the fastq format. The raw data from the sequencing contained a small number of reads with sequencing connectors or of low sequencing quality. To ensure the quality and reliability of data analysis, the raw data required filtration. These raw data include reads with the adapter, reads containing N (which means that base information cannot be determined), and low-quality reads (those with Qphred <=20 base number accounting for more than 50% of the entire read length). The Q20, Q30, and GC contents of the clean data were also calculated. All subsequent analyses were based on high quality analysis conducted by clean data.

We downloaded the reference Genome and Gene Model annotation file directly from the genome website. Then, using 3M HISAT2 v2.0.5, an index of the reference genome was built and the paired end clean reads were matched to the reference genome. New gene prediction was performed with StringTie (1.3.3b) (Pertea et al., 2015). Gene expression level quantification featureCounts (1.5.0-p3) was employed to calculate the readings mapped to each gene. The FPKM of each gene was then calculated according to the length of the gene, and the reading mapped to that gene was calculated.

Differential expression analysis between the two comparison combinations was performed using the DESeq2 software (1.20.0). Benjamini and Hochberg’s method was used to adjust the resulting P-values to control the error discovery rate. DESeq2 found that genes with adjusted P values <=0.05 were assigned as differentially expressed. The corrected P-value and |log2foldchange| were employed as thresholds for significant different-expression. The statistical enrichment of differentially expressed genes in the KEGG pathway was analyzed using the clusterProfiler (3.8.1) software. We utilized a local version GSEA analysis tool http://www.broadinstitute.org/gsea/index.jsp to analyze the GSEA KEGG data set for this species.

### 2.5 Combined transcriptome and metabolome analysis

All the obtained differentially expressed genes (DEGs) and differentially expressed genes metabolites (DEMs) were mapped simultaneously in the KEGG pathway database to obtain co-enriched KEGG pathway information. According to the enrichment pathways of the DEG, the top 10 metabolic pathways for the co-significant enrichment of the DEM and DEG in each comparison group were identified, and GraphPad 8.0.2 was used to generate a histogram. The anthocyanin biosynthesis pathway map was drawn with reference to the KEGG pathway database. The FPKM values of the DEGs and the relative quantitative values of the DEMs involved in the path were standardized and plotted into heat maps to the pathway map.

### 2.6 Weighted correlation network analysis

Weighted total gene network analysis (WGCNA) was conducted with the TBtools software (https://github.com/ShawnWx2019/WGCNA-shinyApp). A total of 4217 DEGs FPKM values were calculated from 24 transcriptome samples (4 time points, 3 replicates). The network type was unsigned and the correlation type was Pearson. R^2^ > 0.85 was selected as the soft threshold standard and the soft threshold was set to 10, the merge cut height was 0.2, the min module size was 30. Correlation analysis was conducted between the content of the two anthocyanins and the obtained modules, and the criterion for selection of candidate modules was correlation coefficient >0.75. The hub gene was screened with gene significance (GS) >0.5 and module membership (MM) >0.8 (MM>0.9 in the turquoise module) as the criteria (Song et al., 2019b).

### 2.7 Analysis of candidate gene correlation and promoter cis-acting elements

All candidate gene sequences were submitted to the PlantTFDB database (http://planttfdb.gao-lab.org/) to predict possible transcription factors. The correlation of the selected candidate genes and anthocyanin components in the co-expression network was analyzed with Cytoscape_V.3.7.1. Plant CARE (https://bioinformatics.psb.ugent.be/webtools/plantcare/html/) was used to predict promoter cis-acting elements of upstream 2000 bp initiation codon of candidate genes. Then, the component prediction information was input into the TBtools (http://www.tbtools.org/) software for mapping.

### 2.8 qRT-PCR analysis

Total RNA from the plants was extracted using a total RNA extraction kit. The integrity of the total RNA was detected by 1.0% agarose gel electrophoresis with 3 ul of the obtained RNA products. Then, cDNA was synthesized by the HiScript^®^ III RT SuperMix for qPCR (+gDNA wiper) kit. The reaction conditions of reverse transcription PCR were 25 °C, 10 min; 50 °C, 15 min; and 85 °C, 5 min. The configuration of the reaction solution needed to be performed on the ice, and after its reverse transcriptional synthesis, the first strand of cDNA was stored in the refrigerator at -20 °C for use. qRT-PCR was performed using the CFX96TM real-time system (Bio-Rad, California, USA) and 2 × TSINGKE^®^ Master qPCR Mix (SYBR Green I) (TSINGKE, Beijing, China) reagents. The PCR primer (S1) was designed using Primer6.0. Using the cDNA as template and β-actin gene as the internal reference, real-time fluorescence quantitative PCR was employed to analyze the expression of related genes. Three biological repeats and technical repeats were observed in each reaction. The amplification procedure involved the following parameters: 95°C, 30 s predenaturation; 95°C, 10 s denaturation, 60°C, 30 s annealing and extension, 40 cycles. The dissolution curve used the instrument default acquisition procedure, using 2^–ΔΔCt^ to calculate the relative gene expression (Livak & Schmittgen, 2001).

## 3 Results and analysis

### 3.1 Changes of anthocyanin content in sweet cherry fruit after shading treatment

**Figure 1:**
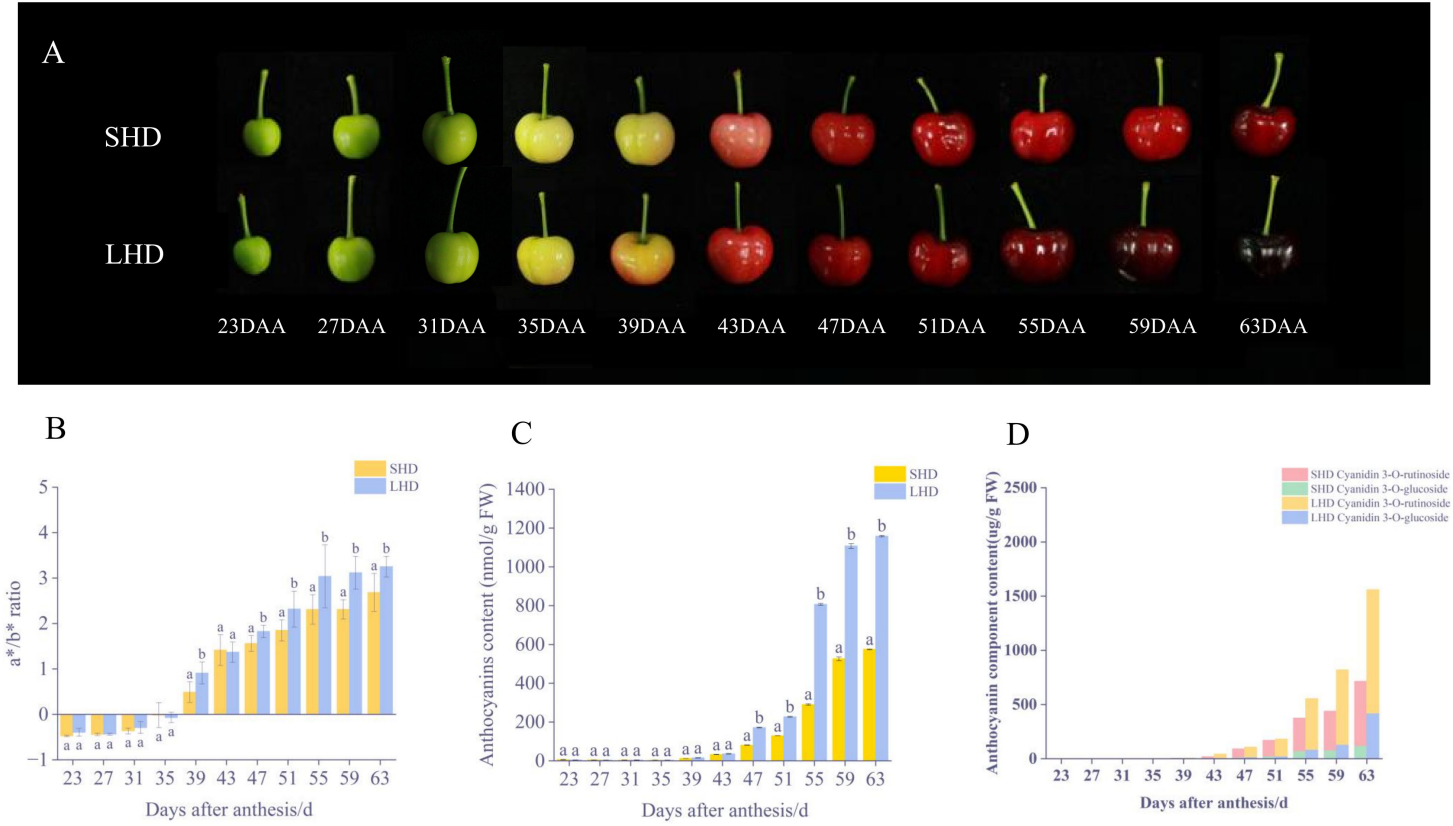
Fruit color changes after shading. **(A)** SHD means Shading “Hong Deng,” LHD means “Hong Deng” under normal light, DAA means days after anthesis during fruit growth and development. **(B)** Changes in fruit color **(C)** Changes in total anthocyanin content **(D)** Changes in anthocyanin component content of fruit.

After the shading treatment, the color of sweet cherry fruits changed significantly. Compared with the fruits under normal light conditions, a lighter red color was observed on the sweet cherry fruits after the shading treatment (**Figure 1A**). No significant difference in the color of sweet cherry fruits was noted in the early stage of shading (23-31 DAA), the a*/b* value was negative, and all fruits were green. At approximately 35 DAA, the sweet cherry fruit entered the whitening stage and began to turn color, and the a*/b* value approached zero. Then, the color gradually turned to deep red, the a*/b* values of the fruits began to change, and the SHD was almost always lower than the LHD till fruit harvest (**Figure 1B**). Consistent with the changes of fruit phenotype and color, the anthocyanin content of the sweet cherry fruits after shading treatment was lower than that of fruits under normal light since 39 DAA. This difference has become significant since 55 DAA, and the difference level has been maintained (**Figure 1C**).

To investigate the effects of shading on anthocyanin components, the said components and contents were determined by HPLC. Two anthocyanins were obtained, cyanidin 3-O-glucoside and cyanidin 3-O-rutinoside (**Figure 1D**). Between them, cyanidin 3-O-rutinoside was the main anthocyanin component in “Hong Deng” sweet cherry, accounting for 65.34% of the total anthocyanin. The results showed that the contents of these two components decreased significantly under the influence of shading treatment, an outcome which was consistent with the changes of total anthocyanin and fruit phenotype.

### 3.2 Metabolome analysis

**Figure 1** showed that after shading treatment, the color and anthocyanin content of sweet cherry fruit changed strongly at 39, 47, 55, and 63 DAA, with substantial differences. To explore the mechanism of influence of light shading on sweet cherry fruit color change, samples from the above four periods were selected for further metabolome and transcriptome analysis.

**Figure 2:**
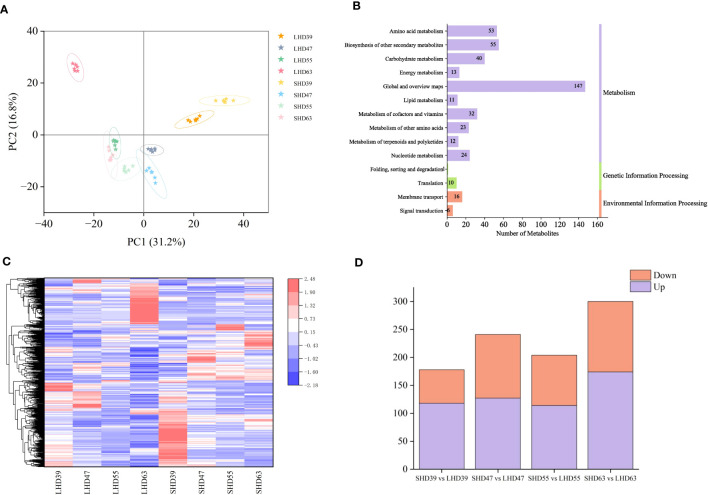
Metabolomic analysis of four periods of fruit after shading treatment. **(A)** PCA analysis of metabolites in different treatment groups at each stage; **(B)** KEGG pathway annotation of metabolites; **(C)** Cluster heat maps of all DEMs in the four periods, with relative levels of metabolites ranging from low (blue) to high (red); **(D)** Numbers of DEMs in the upper (purple) and lower (orange) tones during the four periods.

The metabolome PCA results were shown in **Figure 2A**. A total of 48 samples could be clearly distinguished by the first two principal components, which accounted for 31.2% and 16.8% of the total variability, respectively. Metabolites were divided into 8 groups in total. The repeated samples within each group were closely related, and the distance between the groups was relatively far, indicating the reliability of the metabolome data. Obvious differences were observed in the diagram of sweet cherry samples in the four periods. Fruit samples at 55 and 63 DAA were distributed in the negative end of PC1, and those at 39 and 47 DAA were distributed in the positive end of PC1. That is, fruit samples at the first two periods were significantly different from those at the last two periods. The close distance between SHD63 and LHD55 indicated the strong similarity of metabolites between the two groups. LHD63 was separately distributed in the negative end of PC1 and the positive end of PC2 and was far away from SHD63, indicating that the metabolites were significantly different between the two groups at 63 days after flowering. A total of 1065 metabolites were identified in the metabolome, including 341 positive ions and 724 negative ions. According to KEGG functional annotation, the identified metabolites can be roughly divided into three categories, of which the metabolism group had the largest number of metabolites, reaching 410, accounting for 92.55% (**Figure 2B**).

With threshold VIP > 1.0, difference multiple FC > 1.5 or FC < 0.667, and P-value < 0.05, 678 DEMs were selected. The expression patterns of the DEMs were significantly different among the groups. As shown in **Figure 2C**, the low expression levels of DEMs in the treatment group may be the main cause of the fruit color differences. Comparison of the LHD and SHD samples at different periods revealed that the combinations with the most differentiated metabolites were LHD63 vs. SHD63 (a total of 300 metabolites, 174 up-regulated and 126 down-regulated), and the least differentiated metabolites were LHD39 vs. SHD39. In general, more up-regulated metabolites were observed in the four periods (**Figure 2D**). In summary, shading exerted an effect on the metabolites of sweet cherry fruits in multiple post-flowering periods, and the effect was greatest at 63 days after flowering.

### 3.3 Transcriptome analysis

**Figure 3:**
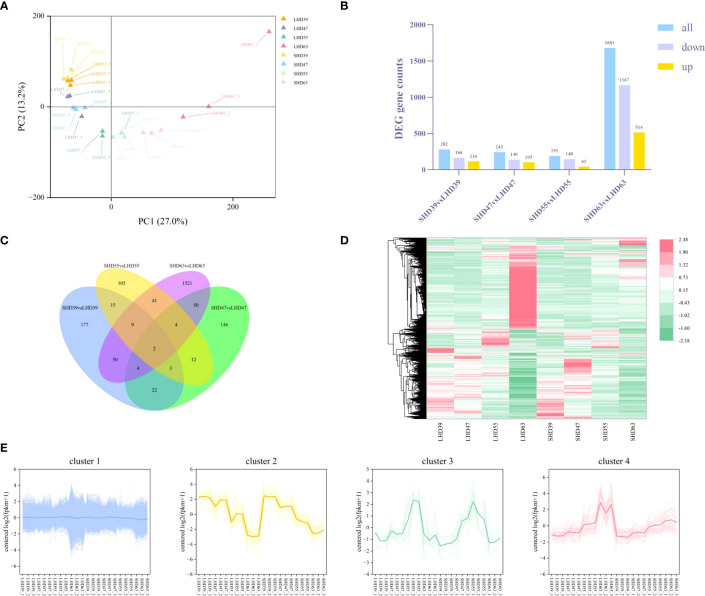
Transcriptome data analysis of fruit at four stages after shading treatment **(A)** PCA analysis of gene expression in different treatment groups at each stage; **(B)** Quantity statistics of the DEGs in the four periods, with yellow representing up-regulation, purple representing down-regulation, and blue representing total DEGs quantity; **(C)** Venn plots of common or unique expression of DEGs within and between comparison groups in each period; **(D)** Cluster heat maps of the expression of all differentially metabolized genes in the four periods, with red representing up-regulation and green representing down-regulation; **(E)** Expression patterns of all DEGs in the four periods, with different colors representing different expression trends, and dark thick lines representing the average expression profiles of all genes in each cluster.

The transcriptomic data of sweet cherry fruit were analyzed by RNA-seq technique after light shading treatment. A total of 1,165,020,240 raw data were generated from the fruit samples over four periods, and 1,145,349,294 high-quality clean readings were obtained after filtering out junction sequences, uncertain readings, and low-quality readings. On average, 95.01% of the clean readings were located on the sweet cherry genome. A total of 45,071 transcripts were used for subsequent analysis (S2). PCA analysis of transcription sample expression was performed (**Figure 3A**). Each sample was clearly well distinguished on the score map, and the focus between each reset was tight, indicating differences in fruit transcripts after shading treatment. Unlike the metabolome results, LHD39, LHD47, SHD39, SHD47, and some LHD55 were on the negative end of PC1, and the rest were on the positive end of PC1. Interestingly, the dispersion between LHD63 and SHD63 was greater in the score map than in the other three periods, suggesting that the shading treatment had a greater effect on the samples in the last period.

The threshold was set to |log2(FoldChange)| >= 1&padj <= 0.05. A total of 4217 DEGs were identified. The DEGs results for comparison among the treated samples in different periods were shown in **Figure 3B**. Similar to the results of the metabolic group, LHD63 vs. SHD63 had the most DEGs among the three groups of comparison, reaching 1681. The down-regulated DEGs outnumbered the up-regulated counterparts. The common or unique DEGs results among the three comparison groups were analyzed (**Figure 3C**). Only 2 genes were differentially expressed in all samples, and most differentially expressed genes were only in LHD63 vs. SHD63 (1521). The FPKM value of the DEGs was used to draw the hierarchical clustering heat map (**Figure 3D**). Significant differences occurred among the treated samples, an outcome which was similar to the results of the metabolic group. Moreover, the expression levels of numerous genes in SHD63 were significantly down-regulated. The H-cluster function was used to divide all DEGs expression patterns into 4 groups, and all samples revealed good repeatability (**Figure 3E**). Cluster 1 contained the largest number of DEGs at 4041, accounting for 96% of the total DEGs. The expression level of DEGs in Cluster 2 was down-regulated with growth and development. In Cluster 3, the DEGs were gently up-regulated to 55 DAA and then down-regulated. In Cluster 4, the expression level of DEGs was up-regulated with fruit growth and development, and the expression level of DEGs in SHD was generally lower than that in LHD. Similar to the variation trend of anthocyanin content, the DEGs in Cluster 4 may be related to the decrease of fruit anthocyanin content after shading.

### 3.4 Transcriptome metabolome combined analysis

#### 3.4.1 KEGG enrichment analysis

**Figure 4:**
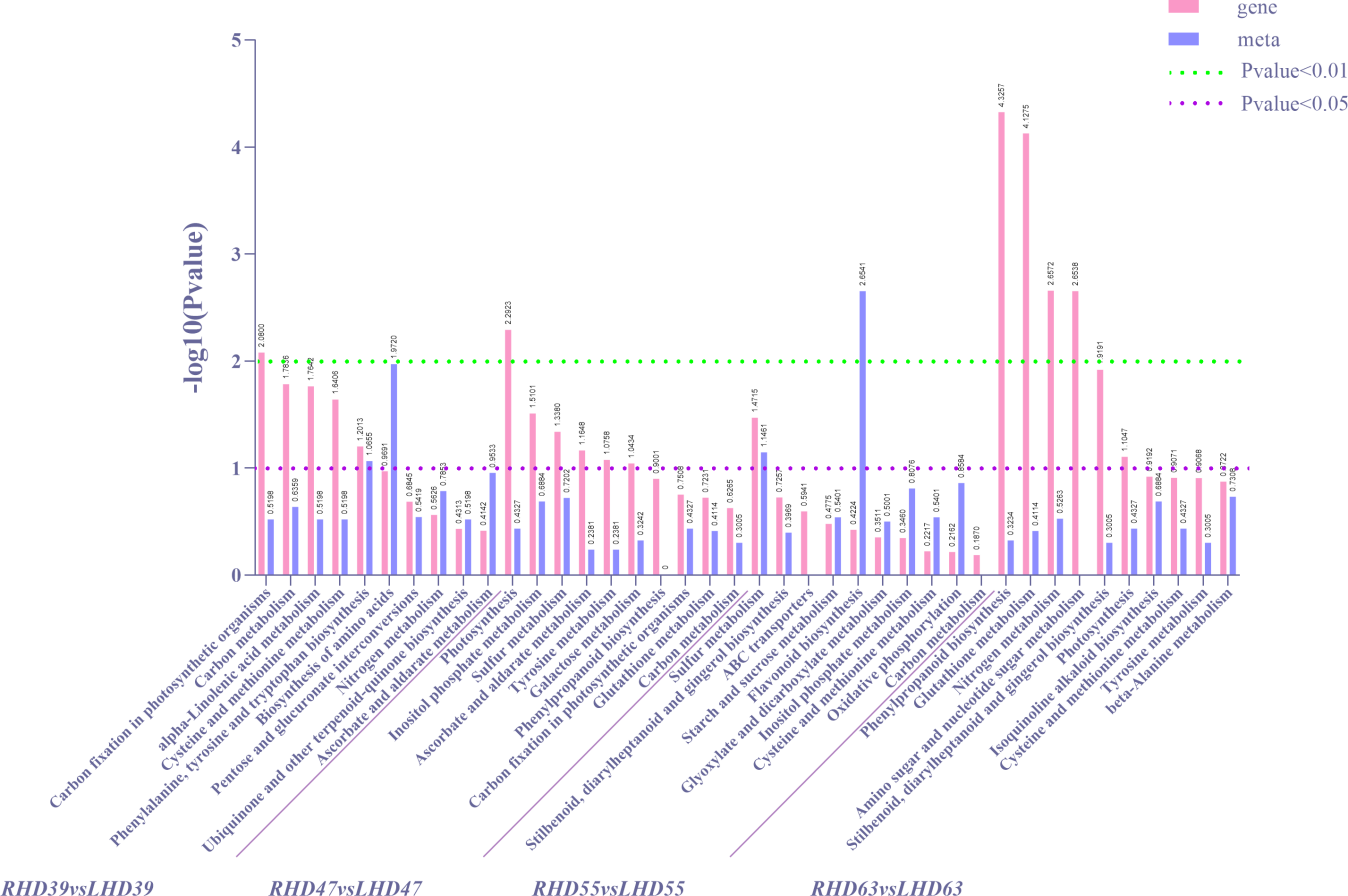
Statistical diagrams of the first 10 KEGG pathways co-enriched by DEMs and DEMs according to DEGs in each comparison group after shading treatment, in which differential gene P-value for enrichment analysis is ≤0.5.

To further determine the metabolic pathways of co-enrichment of DEGs and DEMs, we mapped the top 10 KEGG pathways of co-enrichment of DEGs and DEMs in each comparison group, showing 26 enriched pathways (**Figure 4**). Cysteine and methionine metabolism and carbon metabolism appeared most frequently (3 times). Of all the DEMs enriched KEGG pathways, the most significantly enriched metabolic pathways occurred at 63 DAA. These pathways included phenylpropanoid biosynthesis, glutathione metabolism, nitrogen metabolism, amino sugar, and nucleotide sugar metabolism. Among all the DEMs enriched KEGG pathways, an extremely apparent flavonoid biosynthesis occurred at 55 DAA. In addition, the circadian rhythm-plant pathway was present in the top 20 co-enriched metabolic pathways of both DEGs and DEMs, as detailed in S3. These results indicated a greater effect of shading treatment on structural genes and metabolites related to the upper and middle pathway of anthocyanin biosynthesis (phenylpropanoid biosynthesis and flavonoid biosynthesis in fruit).

#### 3.4.2 Analysis of the anthocyanin biosynthesis pathway

**Figure 5:**
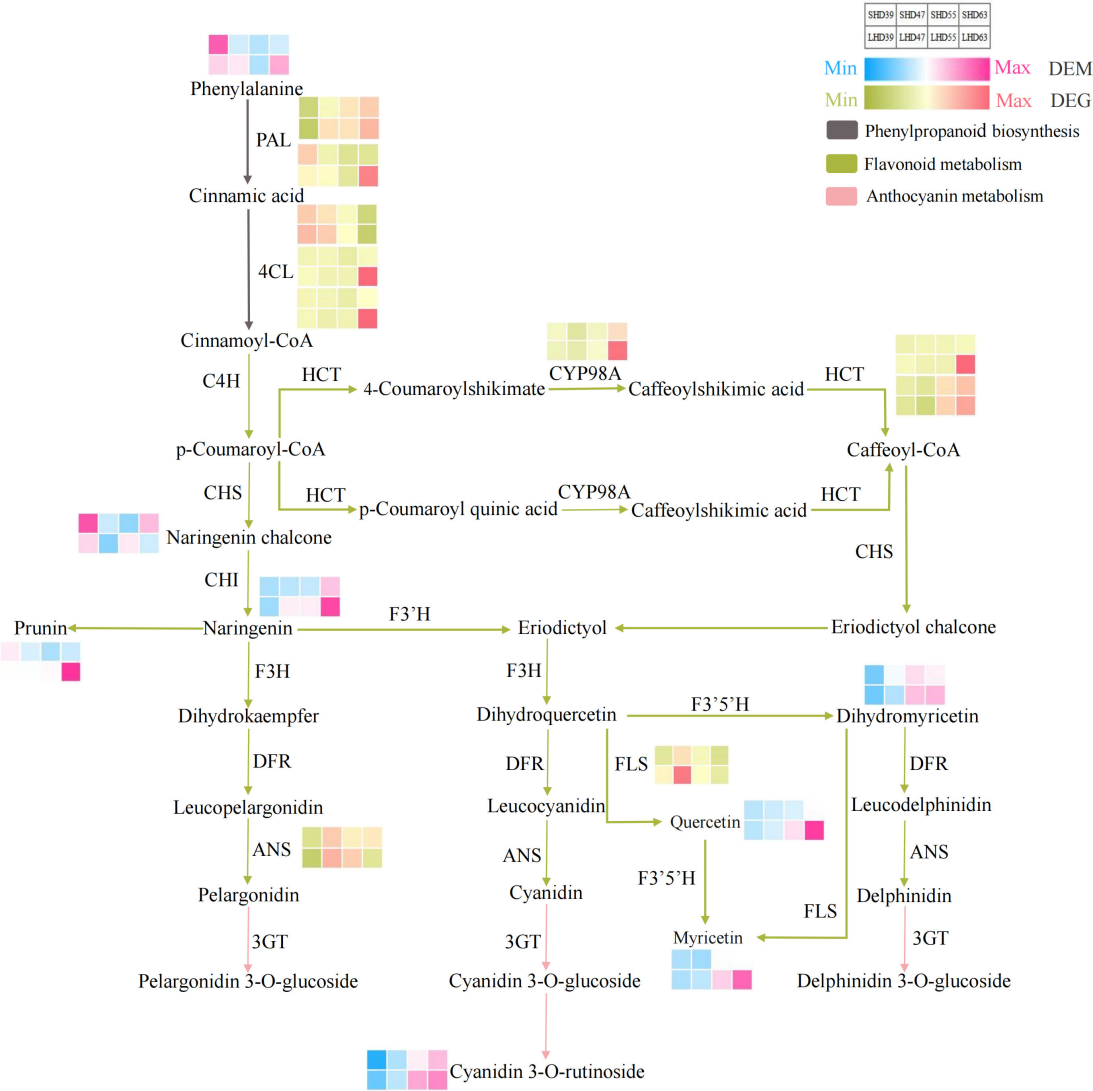
Anthocyanin biosynthesis pathway composed of phenylpropanoid biosynthesis, flavonoid biosynthesis, and anthocyanin metabolism. The red and blue blocks represent the DEMs. Red and green blocks represent DEGs.

To more clearly and visually demonstrate the changes of genes and metabolites in the anthocyanin biosynthesis pathway of fruit after shading treatment, we mapped the said pathway and thermologically mapped the DEGs and DEMs data (**Figure 5**). The pathway mainly included three paths: phenylpropanoid biosynthesis (upstream of anthocyanin biosynthesis), flavonoid biosynthesis (middle stream of anthocyanin biosynthesis), and anthocyanin metabolism (downstream of anthocyanin biosynthesis). The pathway involved 10 DEGs and 8 DEMs.

Phenylalanine was an important precursor substance in phenylpropanoid biosynthesis and the anthocyanin biosynthesis pathway. As the only DEM in the phenylpropanoid biosynthesis pathway, the phenylalanine content increased in the early period of blackout (39 DAA), and decreased in the following three periods compared with the counterpart with normal light. This phenylpropanoid biosynthesis pathway also involved 5 DEGs, 2 *PAL* and 3 *4CL*. The expression of *PAL* was slightly up-regulated at 39 DAA and was down-regulated at other periods. The expression levels of two *4CL* genes were significantly down-regulated at 63 DAA. However, the expression level of one *4CL* gene decreased with fruit development, slightly down-regulated in the early period of anthesis, and up-regulated in the later period (63 DAA).

Flavonoid biosynthesis and anthocyanin metabolism involved 5 DEGs and 7 DEMs. In the flavonoid biosynthesis pathway, the difference of *CYP98A* expression was most significant at 63 DAA. The expression trended of the two *HCT* vary, but both their expression levels were down-regulated after fruit shading. No significant difference occurred in the expression of *ANS* except for slight up-regulation in the fruit at 63 DAA. The expression level of *FLS* decreased after shading and was most obvious at 47 DAA. The DEMs in the flavonoid biosynthesis pathway included prunin, quercetin, naringenin chalcone, naringenin, dihydromyricetin, and myricetin. Except for naringenin chalcone, the contents of the other DEMs in this pathway were decreased by shading treatment at 55 DAA and postflowering 63 DAA, with the highest decrease at 63 DAA. One DEM (cyanidin 3-O-rutinoside) was detected in anthocyanin biosynthesis, and the content was reduced in the shading fruit entity. Which suggested that it may be the main component leading to the lightening of the shading fruit color and the decrease of anthocyanin content.

In general, most of the DEGs showed a down-regulated trend in the shading fruit during the entire biosynthesis pathway. The DEGs in the pathways were more abundant the DEMs, and most of the differences occurred at 63 days postflowering. This finding indicated that the shading treatment had the greatest effect on sweet cherry fruits at 63 days after flowering. The upstream pathway of anthocyanin biosynthesis DEGs, such as the *PAL* and *4CL* genes, changed obviously after sunshade treatment. Then, the contents of anthocyanin biosynthesis precursors such as naringenin decreased in the shading fruit during the three periods. We posited that shading may inhibit the expression of *PAL*, *4CL*, *CYP98A*, and other genes, which led to the reduction of the precursors of anthocyanin synthesis and ultimately causes the decrease of anthocyanin accumulation in the light-shading fruits.

### 3.5 WGCNA analysis of DEGs and anthocyanin components

**Figure 6:**
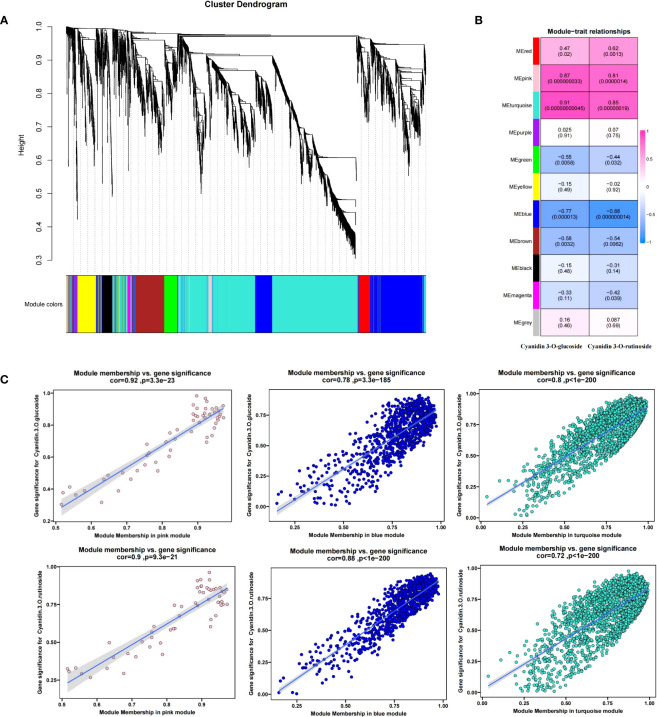
Weighted correlation network analysis modules of all DEGs established after the shading treatment. **(A)** Tree graphs of 4217 DEGs by hierarchical clustering of topological overlapping dissimilarities. **(B)** Heat maps of the correlation between modules and sample traits. Different colors represent different modules (11 in total); the number in the grid indicates the Pearson correlation between the module and the trait, with values ranging from -1 (blue) to 1 (red); and the value in parentheses represents the p-value, such that the smaller the value, the more significant the outcome. **(C)** Gene significance (GS) and module membership (MM) relationships between genes and phenotypic traits (anthocyanin components) in the three candidate modules.

To further determine the molecular mechanism of fruit color change caused by shading, 4217 DEGs were subjected to WGCNA analysis (**Figure 6A**). The 4217 DEGs were divided into 11 modules(S4), with the turquoise module containing the most DEGs (2080) and the gray module with the least (35).

Then, we used the contents of two anthocyanin components (cyanidin 3-O-glucoside and cyanidin 3-O-rutinoside) measured in **Figure 1D** as trait indicators and correlated them with 11 modules. The results were as shown in **Figure 6B**, in which the contents of the two anthocyanin components were highly positively correlated with the turquoise and pink modules and highly negatively correlated with the blue module. The correlation coefficients of the turquoise, pink, and blue modules with the content of cyanidin 3-O-rutinoside were 0.85 (p =1.4 × 10^−9−8^), 0.81 (p =1.9 × 10^−7^), and -0.88 (p =1.4 × 10), respectively. The corresponding correlation coefficients with cyanidin 3-O-glucoside were 0.91 (p =4.5 × 10^−11^), 0.87 (p =3.3 × 10^−6−9^), and 0.77 (p =1.3 × 10). Given the high correlation between the three modules and the components of anthocyanin (r>0.75), a total of 3036 DEGs (2080 turquoise, 55 pink, and 901 blue) were identified from the three modules (S4). The relationship between MM and GS between genes and phenotypic traits (anthocyanin components) in each module was shown in **Figure 6C** (further details are presented in S5). To further narrow down the scope of candidate genes, we set the threshold value MM >0.8 (MM >0.9 in the turquoise module) and GS >0.5 on the basis of the WGCNA. A total of 57 transcription factors such as *bHLH*, *ERF*, *NAC*, *LBD*, and *WRKY*, 5 anthocyanin synthesis structural genes (2 *HCT* and 3 *4CL*) and 4 photoinducable proteins (*RVE1*, *RVE2*, *LHY*, and *FKF1*) were screened, which were chosen as our candidate genes (S6).

### 3.6 Analysis of candidate gene correlation and promoter cis-acting elements

**Figure 7:**
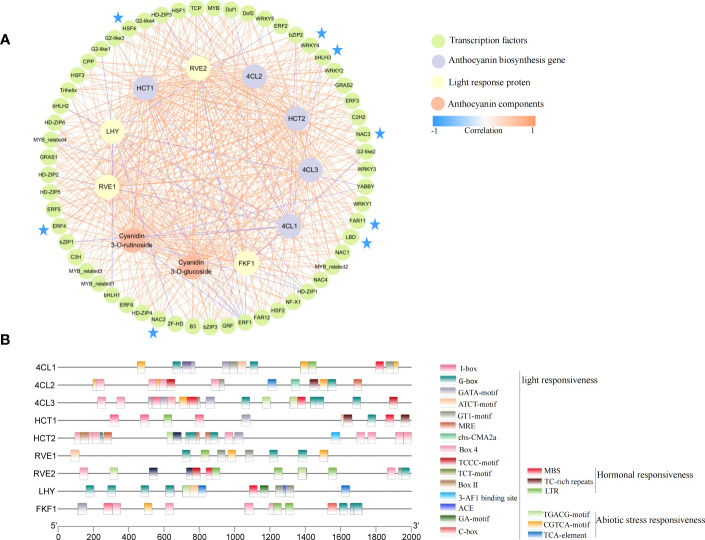
Correlation network and cis-acting elements of candidate genes. **(A)** Correlation network diagram constructed by candidate genes and two anthocyanin components. The colors of the lines represent Pearson correlations (correlations between transcription factors are not included in the figure), from blue (-1) to orange (1). **(B)** cis-acting elements of candidate gene promoters.

To more clearly and intuitively show the relationship between candidate genes and anthocyanin components, we further performed correlation analysis on the obtained candidate genes and two anthocyanin components (S7). Further, the correlations >0.8 and <-0.8 were presented using the Cytoscape software (**Figure 7A**). Among the candidate genes, 4 of 9 genes were in the inner circle (*RVE2*, *LHY*, *HCT1*, and *4CL3*) were clustered in Cluster 4 (**Figure 3E**), an outcome which was consistent with the change trend of total anthocyanin and component contents. Meanwhile, all candidate genes in the figure were closely correlated with anthocyanin components, thereby indicating the credibility of the WGCNA screening results. The 57 transcription factors were divided into a 24-transcription factor family, among which the *bHLH*, *bZIP*, *ERF*, *G2*-like, *LBD*, *HSF*, *NAC*, and *WRKY* transcription factor family members were more closely related to structural genes and anthocyanin components. For example, *bHLH3*, *ERF4*, *LBD*, *NAC2*, *NAC3*, *HSF4*, *FAR11*, and other transcription factors had a correlation coefficient of more than 0.8 with all structural genes and anthocyanin components except for *4CL1*. In addition, transcription factors strongly correlated with structural gene *4CL1* were negatively correlated, but the *4CL2* and *4CL3* were positively correlated with the two components of anthocyanin and most transcription factors. This difference suggested that *4CL* may be the key structural gene that caused the decrease of anthocyanin accumulation in fruits after shading. In the network diagram, the *LHY* gene related to light response was negatively correlated, and most of the transcription factors related to it were highly positively correlated with the content of the two anthocyanin components. Similarly, the MYB-related proteins *RVE1* and *RVE2* associated with light response were significantly positively correlated with *4CL2*, *4CL3*, *HCT1*, *HCT2*, and the two anthocyanin components, suggesting an important role in the reduction of fruit anthocyanin content by shading.

To elucidate the possible regulatory mechanisms of conserved elements in the promoter region of candidate structural genes and photoinducible proteins, we performed cis-acting element analysis on the upstream 2000 bp of these gene sequences (**Figure 7B**). Moreover, 22 elements were screened to participate in light responsiveness, hormonal responsiveness, and abiotic stress responsiveness. Among the elements, the cis-acting regulatory element involved in light responsiveness was the most numerous (15), suggesting that light may play an important role in the regulatory network of these genes.

### 3.7 Fruit phenotype and candidate gene analysis at 63 DAA after light restoration

**Figure 8:**
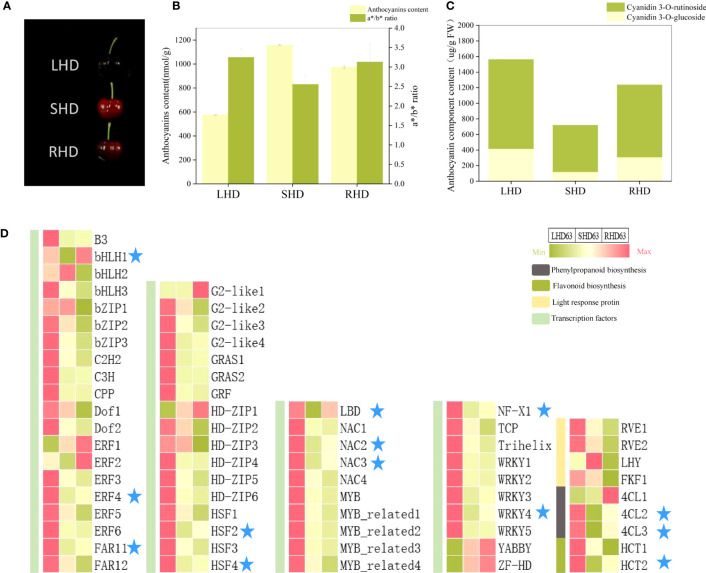
Changes of fruit at 63 DAA after restoring light by removing fruit bags **(A)** changes of fruit phenotype; **(B)** Total anthocyanin content and color changes of fruit; **(C)** Content map of fruit anthocyanin components; **(D)** Expression heat maps of candidate structural genes and regulatory factors in three treated fruits at 63 DAA.

To further narrow down the range of candidate genes that affect the reduction of anthocyanin content in shading fruits, we employed light restoration treatment by removing fruit bags at 55 DAA and harvested fruits at 63 DAA (See S2 for relevant transcriptome information and **Figure S1** for transcriptome PCA). As can be seen from **Figure 8A**, the fruit with restored light exhibited deeper redness, but the fruit color did not return under normal light. The total anthocyanin content, the a*/b* value, and the contents of two anthocyanin components all increased, but were below counterparts for fruits under normal light (**Figures 8B, C**). That is, after shading, the anthocyanin content was between the normal light and shading fruits, and light restoration after shading could not completely restore the anthocyanin accumulation in fruits. Heat maps showed that the expression levels of most transcription factors did not change significantly after light restoration, but the expression levels of transcription factors *LBD*, *bHLH1*, *ERF4*, *FARF11*, *HSF2*, *HFS4*, *NAC2*, *NAC3*, *NF*-*X1*, and *WRKY4* were significantly down-regulated after shading and up-regulated after light restoration (**Figure 8D**). These transcription factors were consistent with the content of anthocyanin components and the expression levels of structural genes *4CL2*, *4CL3*, and *HCT2*.

### 3.8 qRT-PCR verification

**Figure 9:**
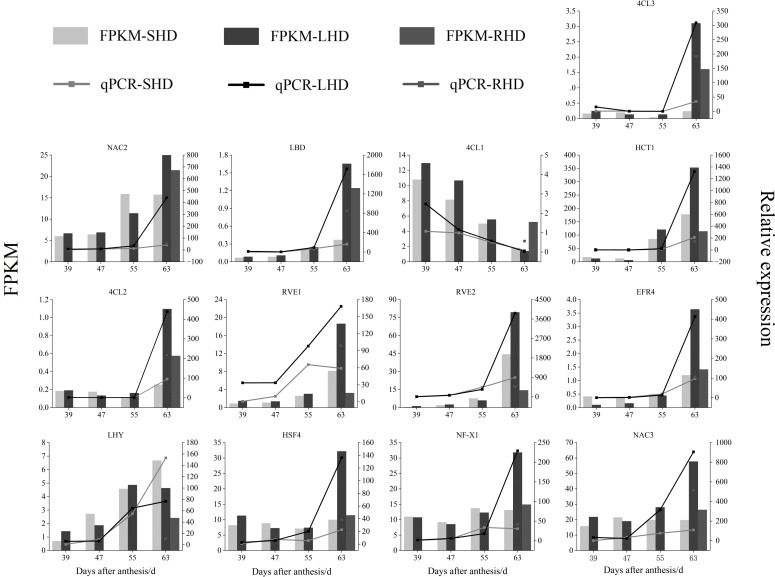
qRT-PCR verification of genes and transcription factors related to the content of anthocyanins in sweet cherry after shading treatment.

Next, we randomly performed qRT-PCR analysis on some of the candidate genes to verify the validity of the transcriptome data **Figure 9**. Although the FPKM values, qPCR relative quantitative values, and specific difference multiples of the selected genes varied in the four stages of sweet cherry fruit, the expression trends of these genes detected by the two methods were the same at the four stages. According to the Pearson’s correlation coefficient (S1), the correlation between the qPCR relative quantitative values and FPKM values ranged from 0.811(*RVE1*) to 0.992(*4CL3*, *EFR4*), indicating the reliability of transcriptome data. The gene expression levels of *4CL2* and *4CL3* in sweet cherry fruits after shading were lower than those in the fruits under normal light, and the expression levels were up-regulated after light restoration. Thus, *4CL2* and *4CL3* may be the key structural genes affecting anthocyanin accumulation. We also verified the expression of *LBD*, *NAC2*, *NAC3*, *ERF4*, and other transcription factors, and their expression trends were consistent with the expression levels of structural genes *4CL2* and *4CL3* and the anthocyanin content. qRT-PCR further proved that structural genes (*4CL1*, *4CL2*, *4CL3*, and *HCT1*) and transcription factors related to anthocyanin biosynthesis showed significant differences among the treated groups, indicating that the reduction of anthocyanin content of “Hong Deng” sweet cherry was related to the upstream structural genes *4CL* and *HCT* and a variety of transcription factors in anthocyanin biosynthesis.

## 4 Discussion

Light is an indispensable environmental factor for plant growth, affecting multiple growth and development processes from seed germination and flowering to fruit coloring. Sweet cherry is a light-loving plant which is widely loved by consumers because of its bright fruit color and juicy taste. The “Hong Deng” sweet cherry is the main red sweet cherry variety in the market at present. In previous studies, considerable evidence indicates that light plays an important role in the coloring of apples, grapes, strawberries, and other fruits (Jiu et al., 2021; Liu et al., 2021; Nguyen et al., 2023). However, until now, few reports are available on the role of light in the coloring of red sweet cherry varieties and its mechanism.

The effect of light on anthocyanin accumulation is achieved mainly through two ways: light quality and light intensity. Currently, the research on the effects of light quality on anthocyanins mainly focus on red light and blue light. In a study applying different light-emitting diodes to strawberries, the anthocyanin content in strawberries increased rapidly after the application of blue light (Zhang et al., 2018b). Tao et al. applied a different light to the Hongzaoyu pear. After 72 h, blue light increased anthocyanin accumulation in pear, but red-light treatment had little effect (Tao et al., 2018). Jian et al. verified that after applying different light intensity to uncolored apples, anthocyanin accumulation increased significantly under medium and high light intensity, and anthocyanin biosynthesis related genes were also upregulated (An et al., 2020a). Under low light intensity (15 µmol/m^2^), Perilla showed a green color, but turned dark red under medium light intensity (180 µmol/m^2^) (Xie et al., 2022).

In this study, the “Hong Deng” sweet cherry fruit was subjected to shading treatment, with the following results: the red color of the fruit became lighter, the anthocyanin content was significantly reduced, and the red color of the fruit was partially restored after the restoration of light. Thus, light played an important role in the color of the “Hong Deng” sweet cherry fruit. In addition to affecting the anthocyanin content of the fruit, the shading treatment also led to a reduction in the weight (**Figure S2**), longitudinal longitude (**Figure S3**) and transverse longitude (**Figure S4**) of sweet cherry fruits, which is consistent with the results of the study on grapes (Melino et al., 2011).Then, we analyzed the sweet cherry fruits in the four selected stage treatment groups by transcriptome and metabolomics, subsequently identifying 678 DEMs and 4217 DEGs. KEGG enrichment analysis showed that various intrinsic qualities of sweet cherry fruits were affected by shading treatment. We focused on the most obvious part of the changes in the fruit phenotype—the fruit color, and found that the sugar metabolism pathway, sugar degradation/gluconeogenesis pathway, phenylpropane synthesis pathway, and flavonoid synthesis pathway related to anthocyanin biosynthesis were significantly enriched. Note that the circadian pathway related to the biological clock of higher plants is also involved, which provides a basis for our follow-up study on the role of light in anthocyanin accumulation in sweet cherry fruits. In the analysis of the anthocyanin biosynthesis pathway, the expressions of *PAL*, *4CL*, *CYP98A*, and *HCT* after shading were significantly down-regulated in the later stage of fruit growth and development (63 DAA). As the middle and upstream genes of the anthocyanin biosynthesis pathway, they have an impact on anthocyanin content. Similar to our findings, the study of A. dahurica revealed that the expression of *4CL* was significantly down-regulated at 90% shade rate (Huang et al., 2022). Likewise, the expression of *4CL* in broccoli was down-regulated after shading treatment (Liu et al., 2020). The transcription level of *PAL* gene in Scutellaria baicalensis was down-regulated under dark conditions (Chen et al., 2010). The expression of *PAL* and *CHS* was also down-regulated with the increase of light intensity in tea plants (Ye et al., 2021). According to Wang et al., light intensity significantly up-regulated the relative expression levels of *CHS*, *FLS*, and *PAL* in bitter malt (Wang et al., 2022a).

WGCNA further narrowed the DEGs range. By setting the MM and GS thresholds, we obtained 66 candidate genes and visualized their correlations. Among the candidate genes, five structural genes (3 *4CL* and 2 *HCT*) were identified as candidate structural genes affecting anthocyanin content in fruit after shading. *4CL* plays an important role in anthocyanin accumulation in fruits. As the upstream gene, different *4CL* isoenzymes can selectively catalyze cinnamic acid, p-coumanyic acid, and other substances to produce the corresponding CoA thioester and then generate anthocyanin through a series of chemical reactions (Wang et al., 2022b). In the study of purple tea, *4CL* was significantly upregulated in the S2_GP (dark grayish purple) stage compared with the S1_RP (reddish purple) and S3_G (medium olive green) stages (Maritim et al., 2021). Similarly, *4CL* expression levels were significantly upregulated in colored Tibetan hulless barley compared to colorless varieties (Xu et al., 2022; Yao et al., 2022). Schulz Dietmar F et al. identified a 4CLSNP marker through GWAS for anthocyanin content and identified it as a candidate gene for influencing anthocyanin concentration (Schulz et al., 2016). *4CL* gene family members are divided into two groups, Types I and II. Type I members are involved in lignin synthesis, and Type II members mostly regulate flavonoid biosynthesis (Wang et al., 2022b). Given the different expression patterns of the three *4Cls* in this study, we speculate that *4CL1* is Type I and *4CL2* and *4CL3* are Type II members. The decrease of anthocyanin content in fruit after shading is more likely related to the down-regulation of *4CL2* and *4CL3* expression levels.

*HCT* is the midstream gene of anthocyanin biosynthesis. The key step of anthocyanin synthesis in potato is the formation of p-coumaryl-CoA, and the down-regulated expression of *HCT* significantly promotes this process (Tengkun et al., 2019). In the study of Cong et al., *HCT* was present in violet-red skin pinellia but was not detected in the pale yellow skin counterpart (Yin et al., 2023). Similarly, *HCT* expression levels in purple testa peanuts were significantly higher than that in pink counterparts. All these evidences indicate that *HCT* also plays an important role in anthocyanin biosynthesis (Li et al., 2022a).

In addition, we identified four light-responsive proteins (*FKF1*, *LHY*, *RVE1*, and *RVE2*) involved in plant circadian pathways and in the regulation of plant photosynthesis and flowering. Note that few investigations have been conducted on plant anthocyanin synthesis (Joo et al., 2017; Yan et al., 2020; Liu et al., 2023). The only existing studies showed that after overexpression of *RVE8* in Arabidopsis thaliana, the structural genes of the anthocyanin pathway such as *4CL* were up-regulated during the day, but this up-regulation was lost at night, suggesting that *RVE8* may play a specific role in the influence of light on anthocyanin biosynthesis (Pérez-García et al., 2015). In the present study, *RVE1* and *RVE2* were highly positively correlated with *4CL2* and *4CL3*, a finding which is consistent with the results of Pérez-García et al. (Pérez-García et al., 2015). *FKF1*, as a photoperiodic blue light receptor (Imaizumi et al., 2003), has recently been linked to cellulose biosynthesis as a negative regulator (Yuan et al., 2019). However, its role in anthocyanin synthesis has not been reported. In this research, *FKF1* was positively correlated with two anthocyanin components, suggesting that *FKF1* may play an important role in the effect of shading on the sweet cherry fruit color, but its mechanism remains unexplored. This work also suggests that *LHY* seems to play a negative regulatory role, a significant negative correlation with *4CL1*, and a weak correlation with other structural genes and anthocyanin components. Although few reports are available on the anthocyanin synthesis pathway of the *LHY* protein, other related pathways such as glucose metabolism and transport, starch degradation, tricarboxylic acid cycle, and phenylalanine metabolism have been examined (Lu et al., 2005; Nakamichi et al., 2009; Graf et al., 2010; Fenske et al., 2015). Therefore, we hypothesize that *LHY* and its related regulators may play a role in another regulatory network, which may affect anthocyanin accumulation by influencing the precursor pathways associated with anthocyanin biosynthesis.

Anthocyanin accumulation in fruits is regulated by a variety of transcription factors. In this study, we obtained 57 potential regulatory transcription factors. Among them, *LBD*, *bHLH*, *bZIP*, *ERF*, *G2-like*, *NAC*, *HSF*, *WRKY*, *NF-X1*, *FAR1*, and other transcription factor families are closely related to the anthocyanin biosynthesis structural genes *4CL2*, *4CL3*, *HCT1*, and *HCT2* and two anthocyanin components. *LBD* is a plant-specific transcription factor which mainly affects the development of lateral vegetative organs such as leaves and roots (Liang et al., 2022). In addition, *LBD* plays an important role in plant resistance to stress and disease (Feng et al., 2022; Jiao et al., 2022). Regarding the role of *LBD* in anthocyanin biosynthesis, *LBD* in Arabidopsis is reported to regulate anthocyanin biosynthesis by inhibiting *PAP1* and *PAP2* (Rubin et al., 2009). Rui-Min et al. showed that Arabidopsis plants post *CsLBD39* overexpression were affected by nitrogen stress, and the contents of nitrate and total anthocyanins were significantly reduced compared with those of the wild type (Teng et al., 2022). The ectopic expression of *MdLBD13* gene in apple inhibited anthocyanin biosynthesis in Arabidopsis by down-regulating the expression of *AtPAP1*, *AtCHS*, *AtCHI*, *AtDFR1*, and *AtUFGT* (Li et al., 2017). Moreover, overexpression of *MdLBD13* also inhibited anthocyanin accumulation in the apple callus (Li et al., 2017).

*bHLH* transcription factors usually affect anthocyanin accumulation by interacting with *MYB* transcription factors, such as in onions, where *AcB2*, which belongs to the *bHLH* IIIf subfamily, interacts with *AcMYB1* to promote transcriptional activation of the anthocyanin biosynthesis structural genes *AcANS* and *AcF3H1* and ultimately increases anthocyanin accumulation (Li et al., 2022c). *MdbZIP44*, a member of the *bZIP* transcription factor family, was identified as a positive regulator of ABA-promoted anthocyanin accumulation (An et al., 2018). The *ERF* family is also involved in transcriptional regulation of anthocyanin biosynthesis. In the study of An et al., *MDERF38* participated in drought stress-induced anthocyanin synthesis by interacting with *MDMYB1* as a positive regulator (An et al., 2020b). *GFR*, a member of the *G2-like* family, further inhibits anthocyanin synthesis genes by inhibiting *bHLH* and *MYB*, thereby negatively regulating anthocyanin accumulation (Petridis et al., 2016). Apple *NAC* family member *MdNAC52* promotes anthocyanin accumulation by binding to promoters of *MdMYB9* and *MdMYB11* (Sun et al., 2019). Similarly, *MdWRKY11*, a member of the *WRKY* family of red flesh apples, is involved in anthocyanin accumulation by influencing the *MYB* transcription factor and the photoresponse factor *MdHY5* (Liu et al., 2019b). FAR1 transcription factor may also be involved in anthocyanin regulation in citrus (Jin et al., 2023). In conclusion, these transcription factors regulate the accumulation of anthocyanins in plants by directly or indirectly affecting the structural genes of anthocyanin biosynthesis.

To analyze the possible mechanism of action between transcription factors and structural genes, we conducted promoter cis-acting elements analysis for the first 2000 bp of the structural genes and photoinducable protein sequences screened in this study. We found that all these genes contained light response elements. Among them, G-box, a motif that has been shown to be necessary and sufficient to induce light-responsive transcription of certain genes (Giuliano et al., 1988), has played an important role in the regulation of photoinduced anthocyanin biosynthesis in pear (Liu et al., 2019a). G-box has been found to exist in candidate structural genes, and the number of G-box motifs is highest in *4CL3* and *HCT2*. In previous studies, promoter sequences of the potato *4CL* gene family members all contain photoresponsive elements. UV stress can promote the up-regulation of *St4CL6* and *St4CL8* but inhibit the expression of *St4CL5* (Zhong et al., 2022). Studies in tea tree also showed that the promoters of two *4CL* genes, *Cs4CL1* and *Cs4CL2*, contained 9 kinds of light response elements, and *Cs4CL2* was induced by UV-B (Li et al., 2022b). In parsley, both *Pc4CL*-1 and *Pc4CL*-2 were transcriptionally activated by UV irradiation (Douglas et al., 1987). This finding presents a novel idea. Thus, we posit that the shading treatment in the present study may affect the binding of regulatory proteins to the G-box motif in the *4CL* and *HCT* promoters and then affect the transcription of these structural genes, ultimately influencing anthocyanin accumulation.

To further narrow down the range of candidate genes that affect the decrease of anthocyanin content in fruits after shading, we removed the fruit bags at 55 DAA to restore light and harvested the fruits at 63 DAA. The transcriptome data showed that the expression levels of several transcription factors and structural genes discussed above were changed to varying degrees after light restoration. Specifically, *LBD*, *NF-X1*, *FAR1*, *ERF4*, *NAC2*, *NAC3*, *HSF4*, and *WRKY4* showed the same change trend as *4CL2*, *4CL3*, and *HCT2*, and their expressions were up-regulated after light restoration (*LBD* was the most strongly up-regulated), which further confirmed the possible positive regulatory role of these genes in anthocyanin biosynthesis. However, the expression levels of *FKF1*, *RVE1*, and *RVE2* proteins decreased at 63 DAA after light restoration. If these light-responsive proteins act as positive regulators in the light-induced anthocyanin biosynthesis of red sweet cherry, we speculate that the regulatory effects of these proteins on anthocyanin biosynthesis may be time-sensitive. In other words, the expression levels of *FKF1*, *RVE1*, and *RVE2* may be up-regulated within a short time after bag-removal and affect the expression levels of related transcription factors (*LBD*, *ERF4*, *NAC2*, *NAC3*, etc.) and structural genes (*4CL* and *HCT*), resulting in the fruit color darkening after light restoration.

In this study, the potential regulatory genes for photoinduced anthocyanin biosynthesis of sweet cherry were screened by combining transcriptome and metabolome analysis. The results showed that light shading had a negative effect on anthocyanin accumulation and changed the expression levels of several related genes in red light sweet cherry. *4CL2*, *4CL3*, and *HCT2* may be the key structural genes affecting anthocyanin content, and their expression levels may be regulated by transcription factors such as *LBD*, *NF*-*X1*, *FAR1*, *ERF4*, *NAC2*, *NAC3*, *HSF4*, *WRKY4*, *FKF1*, *LHY*, *RVE1*, and *RVE2*. However, the regulatory network of plant growth is intricate, and its specific regulatory modes still need further investigation.

## Data availability statement

The datasets presented in this study can be found in online repositories. The names of the repository/repositories and accession number(s) can be found below: https://ngdc.cncb.ac.cn/gsa/browse/CRA011359, CRA011359.

## Author contributions

YZ and RG: designed the whole experiments. YZ and CC: Data determination. YZ, CC, YC, WeT, GK, WaT, HC: analyzed the data. YZ:wrote the manuscript. RG: supervision. All authors read and approved the final manuscript.
