# Supplementary figures and images for: Potential regulatory genes of light induced anthocyanin accumulation in sweet cherry identified by combining transcriptome and metabolome analysis
Source: Front Plant Sci. 2023 Aug 16;14:1238624. doi: 10.3389/fpls.2023.1238624 (PMC10469515; doi:10.3389/fpls.2023.1238624)

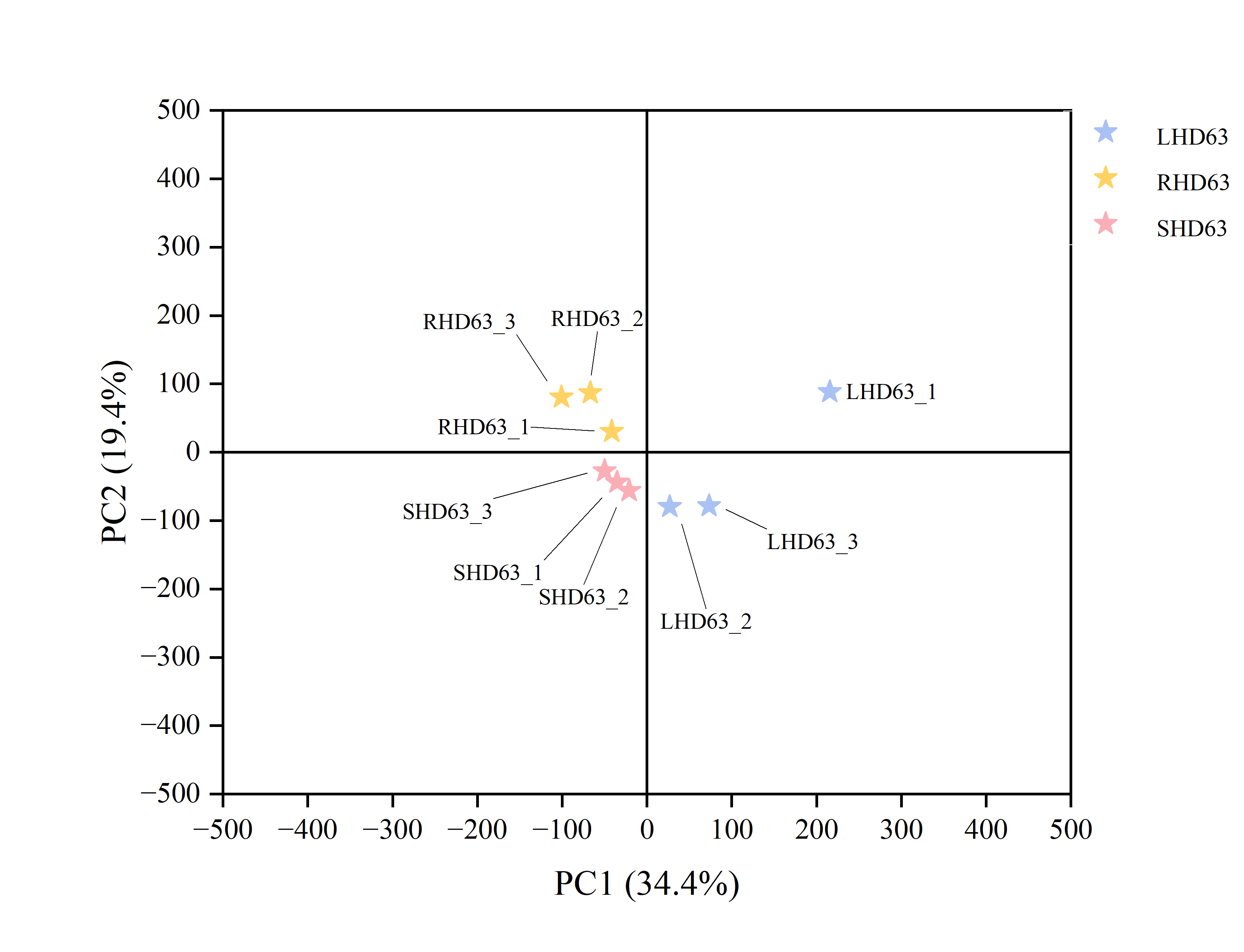

Supplement: Supplementary file 1 [file Image_1.jpeg]

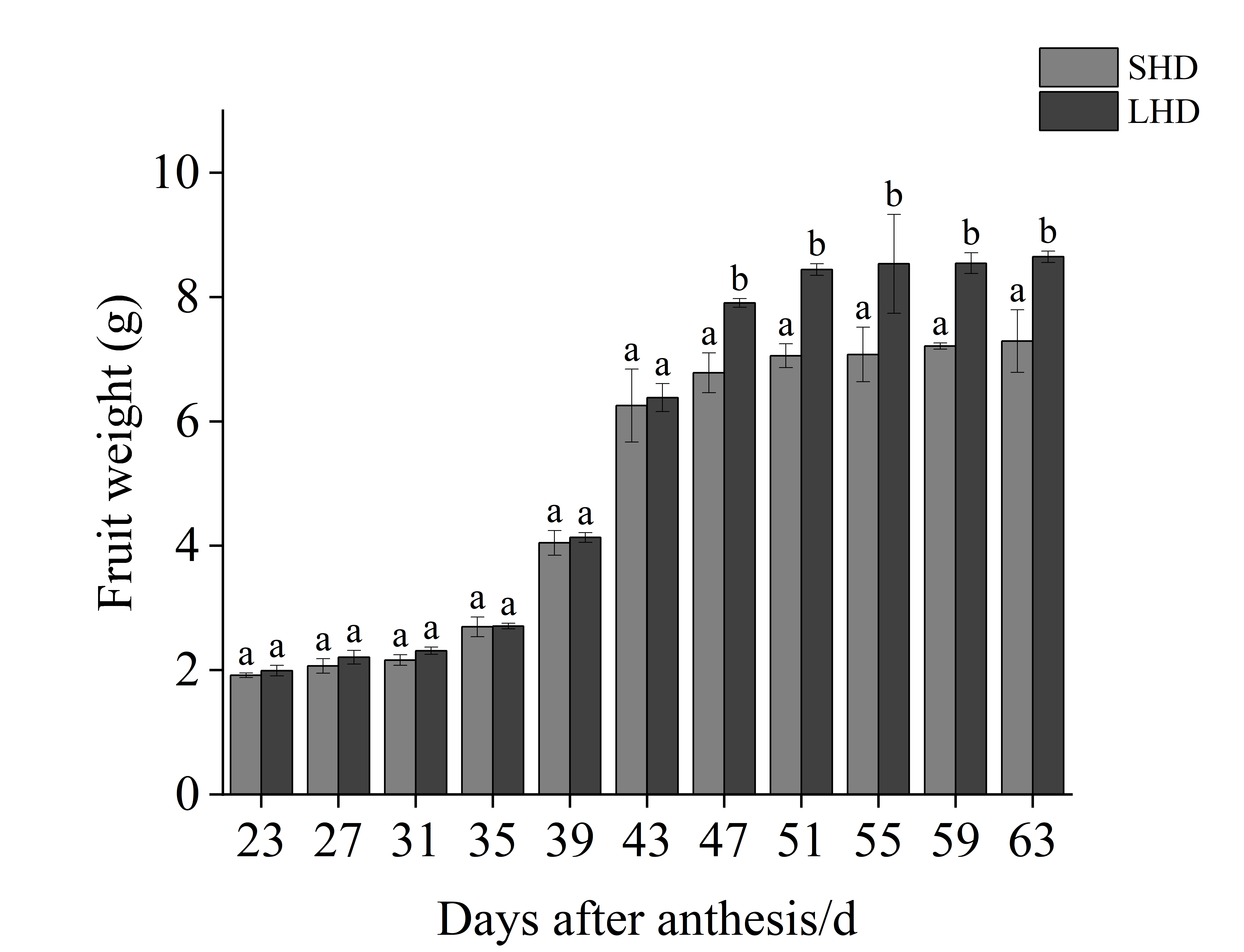

Supplement: Supplementary file 2 [file Image_2.jpeg]

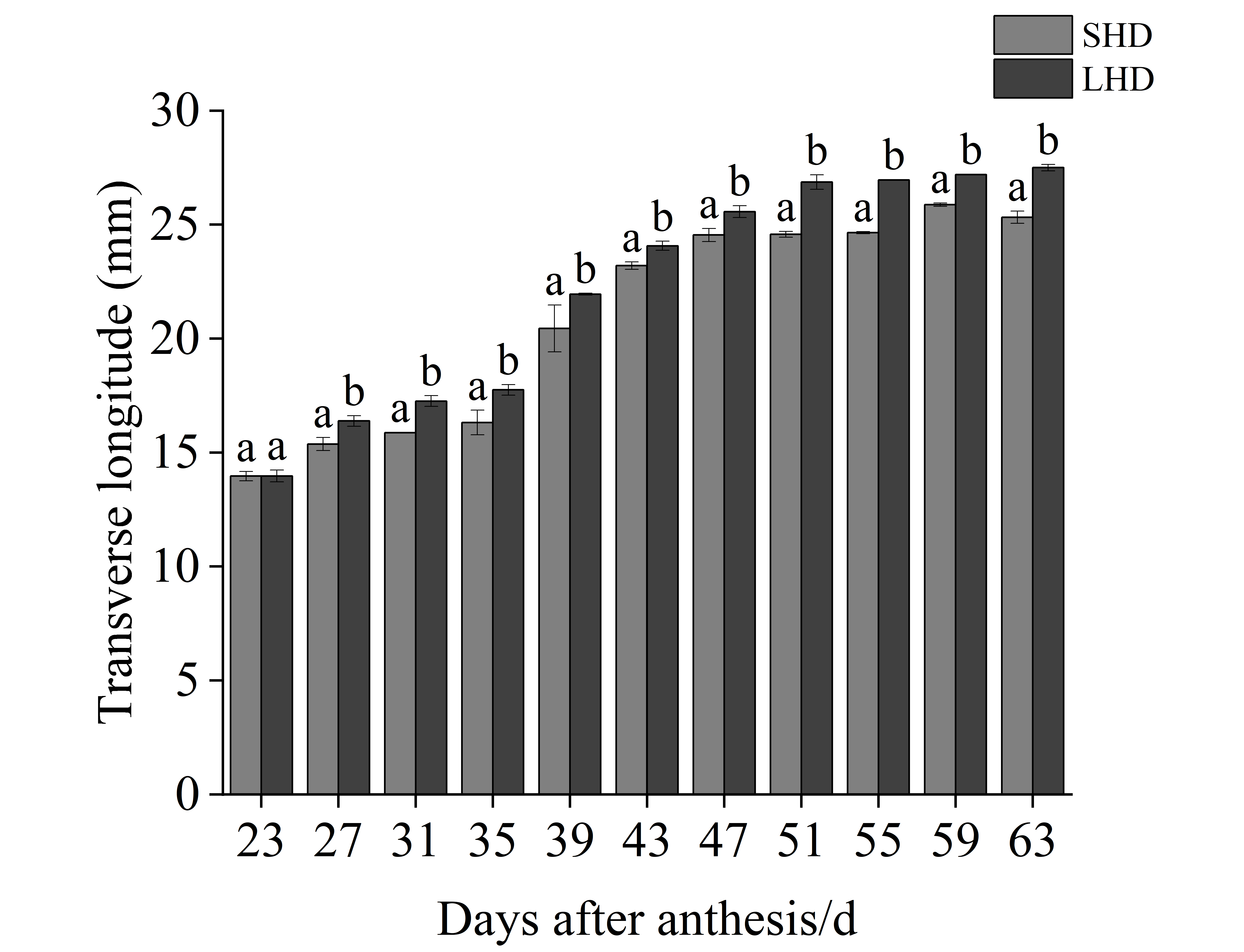

Supplement: Supplementary file 3 [file Image_3.jpeg]

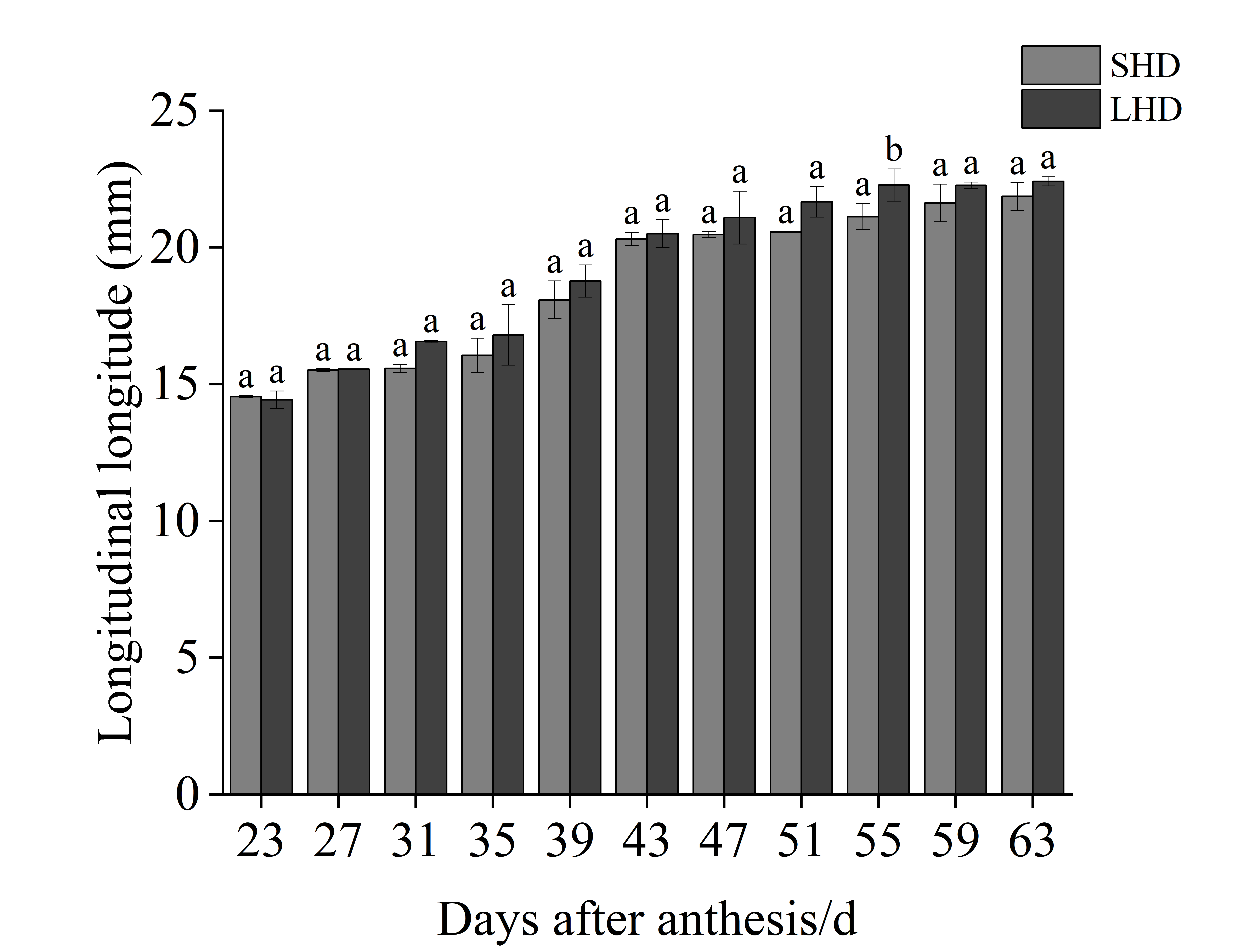

Supplement: Supplementary file 4 [file Image_4.jpeg]
